# Supplementary material for: Vaccine against Streptococcus suis Infection in Pig Based on Alternative Carrier Protein Conjugate
Source: Vaccines (Basel). 2022 Sep 27;10(10):1620. doi: 10.3390/vaccines10101620 (PMC9612095; doi:10.3390/vaccines10101620)
Supplement: Supplementary file 1 [file vaccines-10-01620-s001.zip › vaccines-1921591-supplementary.pdf]

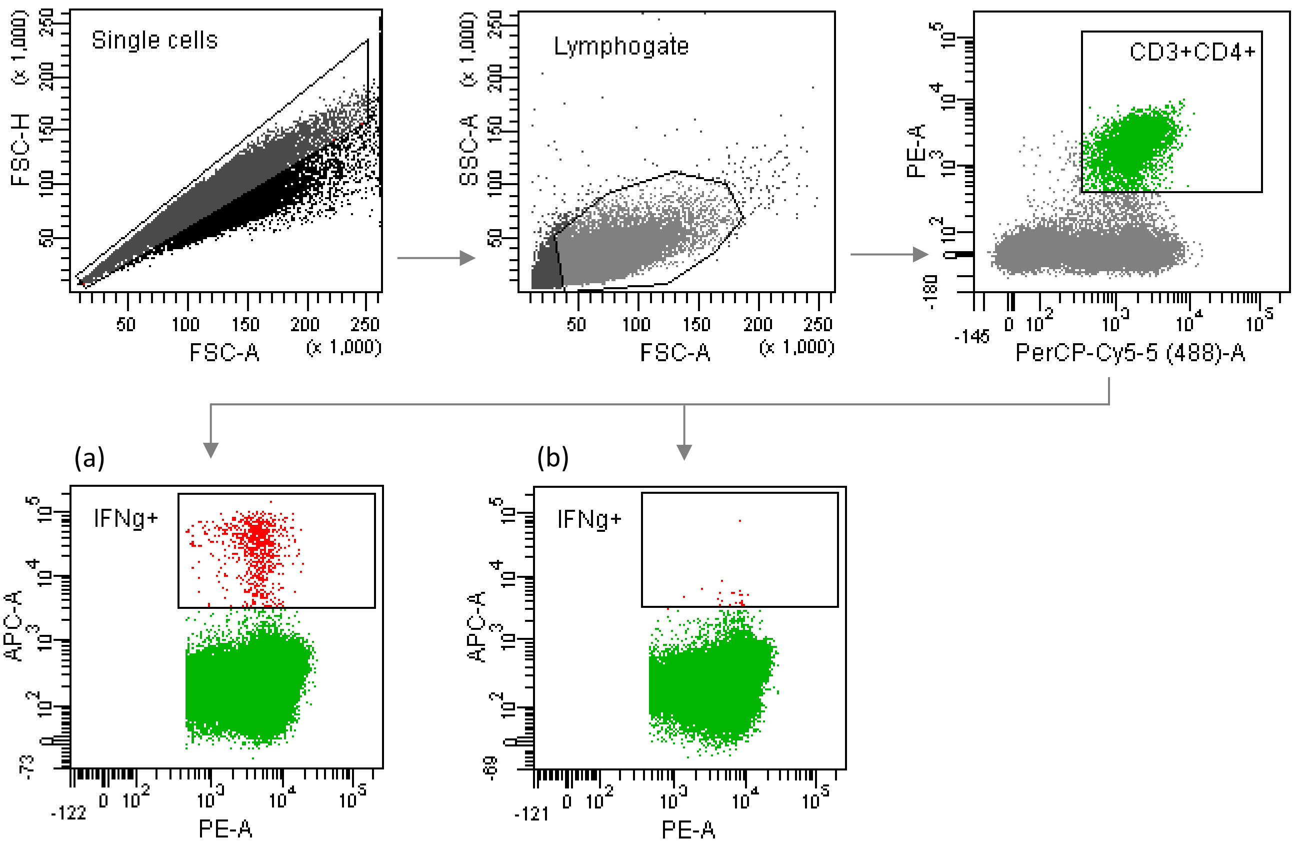

Figure S1. Percentage of IFNγ was analyzed from all CD3+CD4+ cells. Percentage of CD4+IFNγ+ from cCPS-stimulated sample (a) and percentage of CD4+IFNγ+ from un-stimulated sample (b) were analyzed as shown in gating strategy.
